# Determinants of Non-Participation in Population-Based Breast Cancer Screening: A Systematic Review and Meta-Analysis

**DOI:** 10.3389/fonc.2022.817222

**Published:** 2022-03-02

**Authors:** Lilu Ding, J. Wang, M. J. W. Greuter, M. Goossens, Guido Van Hal, Geertruida H. de Bock

**Affiliations:** ^1^Department of Epidemiology, University of Groningen, University Medical Center Groningen, Groningen, Netherlands; ^2^Department of Radiology, University of Groningen, University Medical Center Groningen, Groningen, Netherlands; ^3^Department of Robotics and Mechatronics, University of Twente, Enschede, Netherlands; ^4^Center for Cancer Detection, Flanders, Belgium; ^5^Department of Social Epidemiology and Health Policy, University of Antwerp, Antwerp, Belgium

**Keywords:** breast cancer, mammography, mass screening, participation, determinant

## Abstract

**Background:**

Breast cancer (BC) screening can be performed in a screening program (BCSP) or in opportunistic screening. The existing reviews on the determinants of non-participation depend on self-reported data which may be biased. Furthermore, no distinction was made between the probably different determinants of both screening strategies.

**Objective:**

To find the determinants of non-participation in BCSP by means of a meta-analysis.

**Methods:**

PubMed, Embase, and Web of Science were searched for observational studies which quantified factors associated with non-participation in BCSP in a general population. Studies on opportunistic screening and studies using self-reported data were excluded. A random-effect model was used to calculate pooled odds ratios (ORs) and 95% confidence intervals (CIs). Potential sources of heterogeneity were explored by stratification of the results.

**Results:**

Twenty-nine studies with in a total of 20,361,756 women were included. Low income (OR: 1.20, 95% CI: 1.10–1.30), low education (OR: 1.18, 95% CI: 1.05–1.32), living far from an assigned screening unit (OR: 1.15, 95% CI: 1.07–1.24), being immigrant (OR: 2.64, 95% CI: 2.48–2.82), and having a male family doctor (OR: 1.43, 95% CI: 1.20–1.61) was associated with higher non-participation in screening. Reminders sent to non-attenders and estimations of ORs (adjusted or not) partly explained substantial heterogeneity.

**Conclusion:**

In this meta-analysis excluding studies on the non-participation in opportunistic screening, or with self-reported data on non-participation, the well-known determinants for non-participation are still significant, but less strong. This analysis only supports the relevance of meta-analysis of studies with registered non-participation in a BCSP.

**Systematic Review Registration:**

PROSPERO, CRD42020154016.

## Introduction

Breast cancer (BC) is the most frequent cause of female cancer death (1) and accounts for an estimated 11.6% of the total cancer deaths worldwide in 2018 (2). The risk of BC death can be reduced by 20% when BCs are detected at early stages by mammography screening (3). A breast cancer screening program (BCSP) with mammography is therefore widely advised for early BC detection (4). Compared with opportunistic BC screening that provides mammography screening on request of women and depends on the healthcare insurance of women (5), a BCSP is population-based and characterized by actively inviting women to BC screening and comprehensive quality assurance activities such as training and audit of the program (6).

Sufficiently high participation is a crucial element for the success of a BCSP. To ensure the performance and the public health impact of the population-based BC screening program, a 70% participation rate is recommended as an acceptable level of participation by the European guidelines for quality assurance in breast cancer screening and diagnosis (6). While European countries had one of the earliest provided BCSP since 1986 (7, 8), the average level of screening participation in Europe was only 57.4% (range 27.4–82.6%) in 2016 (9). Outside Europe, BCSP has an even lower participation rate ranging from 18.1 to 55.3% in 2016 (9, 10).

There are several systematic reviews on determinants of non-participation in BC screening (5, 11–17). Main determinants for non-participation reported thus far are low income, low education, living in a rural area, being an immigrant, and comorbidity. However, these systematic reviews either combined results from BCSP and opportunistic screening settings, or included self-reported non-participation in BC screening. Studies showed that the self-reported non-participation tend to be over-reported by women (18, 19). Determinants of non-participation have not been reviewed and meta-analyzed specifically for registry data from BCSP. Therefore, we aimed to evaluate determinants of screening non-participation with registry based studies, namely, recent publications with meta-analysis.

## Methods

We conducted a systematic review according to the guideline of the Cochrane Collaboration (20) and reported the results following the guideline of the Preferred Reporting Items for Systematic Reviews and Meta-Analyses (PRISMA) (21). The protocol of this systematic review was registered on PROSPERO (record number CRD42020154016).

### Search Strategy and Study Selection

Articles were identified in PubMed, Embase, and Web of Science. All databases were searched for studies published between January 1, 2010, and October 31, 2021. The search start year of 2010 was selected to balance the recency and efficiency as the screening guidelines and macro-social demographic factors changed over the last years. A detailed search strategy per database can be found in the **Supplementary File**. Additionally, the reference lists in the retrieved articles were searched to identify additional studies.

### Inclusion and Exclusion Criteria

Observational studies were included if they examined the relationship between determinants and the non-participation of a BCSP with mammography and were published in English. The non-participation in a BCSP was defined as the proportion of women who did not participate in the mammography screening within a required screening interval of a BCSP among all invited women. Studies were excluded in one of the following cases: the non-participation in an opportunistic BC screening was studied, the screening participation data were collected through self-reporting of participants, and determinants of screening re-attendance were studied. Besides, case reports, letters, comments, editorials, reviews, and conference abstracts were excluded.

Two reviewers (LD, JW) independently conducted the screening of articles first based on title and abstract and then based on full text. Disagreements encountered were resolved through discussion or adjudicated by a third reviewer (GB).

### Data Extraction and Quality Assessment

Two reviewers (LD, JW) independently extracted data regarding study characteristics (author, publication year, country, screening period and population size, determinants of non-participation, and non-participation rate), organizational characteristics of a BCSP (targeted age, screening interval, follow-up strategy and payment of screening), and odds ratio (OR) of the determinants of non-participation. In case the association represents determinants and screening participation, ORs were recalculated by 1/OR. The corresponding 95% confidence intervals (CIs) were recalculated likewise. If available, adjusted odds ratios (ORs) with 95% CIs were extracted. Otherwise, crude ORs and 95% CIs were extracted or calculated based on the number of screening non-attenders and attenders for each determinant (22). If multiple articles were published with data of the same study population, determinants in the article that reported the OR with the most adjusted model or with the largest sample size was selected. However, if the articles that were published from the same study reported multiple unique ORs for different determinants of screening non-participation, they were all included for the different determinants in the meta-analysis. The quality of the included studies was assessed with the critical Appraisal tool for Cross-Sectional Studies (AXIS) (23). The AXIS checklist intends to assess the validity and bias of cross-sectional studies with 20 questions in five domains, namely, study aim, methods, results, discussion, ethical approval, and funding (see **Table S1**).

### Statistical Analysis

Determinants reported as categorical variables were dichotomized, in which the reference category applied in the study was tested against the other categories combined. OR and the corresponding 95% CI between the reference group and combined category was calculated (22). Estimates of continuous variables were included or if needed, transformed from regression coefficients to ORs and 95% CIs. The inconsistency (I^2^) test was used to measure heterogeneity. Under the assumption of heterogeneity, a meta-analysis using a random-effects model was performed for each determinant for which at least three studies were available. For each determinant, a stratified analysis was performed to explore the sources of heterogeneity. Based on the published studies, the factors that were related to the heterogeneity of non-participation were considered as stratified factors which included the type of invitation (any invitation or the first invitation), the interval of screening (24 months or 36 months), study region (North America, Europe or Asia), payment of screening (free or co-payment), reminders for non-attenders (yes or no) and estimations of ORs (adjusted or not). For the dichotomized determinants, the heterogeneity caused by the different categorization of determinants was also explored in the stratified analyses in which studies applied different categorizations were pooled separately. A sensitivity analysis was performed to evaluate the robustness of the pooled estimates by sequentially removing each study (24). Publication bias was estimated using a funnel plot and assessed formally with Begg’s test. All statistical analyses were performed with Stata 14 (StataCorp LP, College Station, TX, USA).

## Results

### Characteristics of the Included Studies

**Figure 1:**
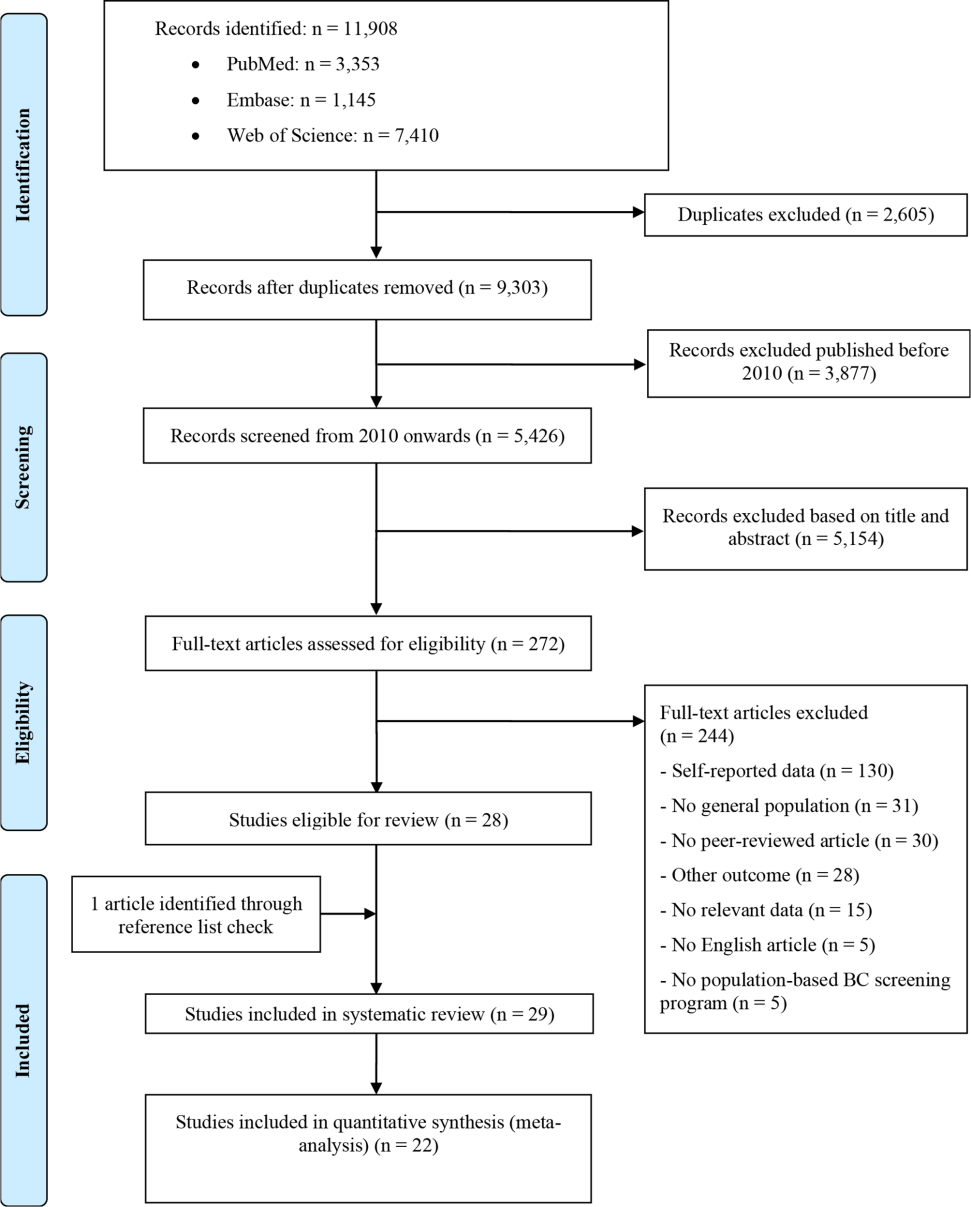
Flow chart of the study selection.

A total of 11,239 studies were identified in the search. A review of 5,299 titles and abstracts and 272 full texts resulted in 29 studies for the systematic review (**Figure 1**). Studies were from 11 countries where a BCSP was established (Canada, Denmark, Sweden, Norway, the United Kingdom (UK), France, Germany, the Netherlands, Israel, South Korea, and Australia). The total number of women in the included studies was 20,361,756, of which 14,944,899 were included in the meta-analysis. Three large studies from Asian countries (Korea, Israel, and Australia) took half of the total population size. The rest of the included women were of European or Canadian origin. The characteristics of the included studies are summarized in **Table 1** (25–53). Twenty-two studies were included in the meta-analysis (**Table S3**) (26–28, 31–41, 44, 46, 47, 49–53).

**Table 1:** Characteristics of the included studies.

Author, year	Country, screening year	Data source	Number of women	Target screening age, years	Screening interval, month	Fully subsidized	Reminder for all non-attenders	Non-participation %	Meta-analyzed determinants^*^
Hellmann (25)	Denmark, 1993–1999	Copenhagen mammographic screening register; Danish Diet, Cancer, and Health cohort baseline data	5,134	50–64	24	yes	yes	10.8	–
Vahabi (26)	Canada, 2010–2012	Citizenship and Immigration Canada database; Ontario Cancer Registry; Ontario BC Screening Program database	1,407,060	50–69	24	yes	no	36.0	Income level, place of residence, gender of family physician
Jack (27)	UK, 2006–2009	London Quality Assurance Reference Centre database	159,078	50–52	36	yes	no	39.0	Income level
Woods (28)	Canada, 2013–2014	Screening Mammography Program of British Columbia database; BC Cancer Registry database; Medical Services Plan physician payment file; Citizenship and Immigration Canada database	537,783	50–69	24	yes	yes	49.7	Age of women, income level, number of comorbidities
Woodhead (29)	UK, 2010–2013	Clinical Record Interactive Search Lambeth DataNet	26,010	50–70	36	yes	no	44.2	–
Price (30)	UK, 2000–2002	Warwickshire, Solihull and Coventry Breast Screening Service database	18,730	50–70	36	yes	no	20.7	–
Guillaume (31)	France, 2003–2012	French cancer screening management database	64,102	50–74	24	yes	yes	49.9	Age of women, income level, distance to an assigned screening unit
Vigod (32)	Canada, 2002–2004	Ontario Breast Screening Program; Ontario Health Insurance Plan; Ontario Cancer Registry; Canadian Community Health Survey database	1,403	50–68	24	yes	no	39.2	Education level, number of comorbidities, marital status
Renshaw (33)	UK, 2004–2007	London Quality Assurance Reference Centre database	742,786	50–70	36	yes	no	37.9	Age of women, income level
Ouédraogo (34)	France, 2010–2011	French cancer screening management database	13,565	50–74	24	yes	yes	47.5	Age of women, income level, place of residence
St-Jacques (35)	Canada, 2006–2008	Information system of the Quebec BC Screening Program; comprehensive Quebec Health Insurance Plan database	833,856	50–69	24	yes	yes	47.9	Age of women, income level, place of residence, distance to an assigned screening unit
Jensen (36)	Denmark, 2008–2009	Central Denmark regional cancer screening administrative database; Danish Cancer Registry; Statistics Denmark	144,264	50–69	24	yes	no	21.1	Income level, distance to an assigned screening unit, immigration status
Le (37)	Norway, 1996–2015	Cancer Registry of Norway’s databases; Statistics Norway	885,979	50–69	24	no	yes	26.0	Age of women, income level, education level, marital status, immigration status,
Zidar (38)	Sweden, 2011–2012	Radiological Information System; Statistics Sweden; Public Health Agency of Sweden; National Board of Health and Welfare; Swedish Social Insurance Agency	52,541	50–74	24	no	no	19.0	Age of women, distance to an assigned screening unit
Jensen (39)	Denmark, 2008–2009	Central Denmark regional cancer screening administrative database; Danish Cancer Registry; Statistics Denmark; Danish National Patient Registry; Danish Psychiatric Central Research Register	144,264	50–69	24	yes	no	21.1	Age of women, education level, number of comorbidities, marital status
McDonald (40)	Canada, 1996–2011	Medicare Decision Support System; BC screening service database; Provincial Cancer Registry; Vital Statistics database of the Province of New Brunswick, Canada	91,917	50–69	24	yes	yes	45.0	Income level, place of residence, distance to an assigned screening unit, education level, marital status
Berens (41)	Germany, 2010–2011	Routine data from screening units and population registries in Duisburg, Bielefeld, Paderborn, Hamburg, and Berlin, Germany	423,649	50–69	24	yes	no	50.8	Age of women
Jensen (42)	Denmark, 2008–2009	Central Denmark regional cancer screening administrative database; Danish Cancer Registry; Statistics Denmark; Danish National Patient Registry; Danish Psychiatric Central Research Register	4,512	50–69	24	yes	no	14.9	–
Jensen (43)	Denmark, 2008–2009	Central Denmark regional cancer screening administrative database; Danish Cancer Registry; Statistics Denmark; Health Survey database in the Central Denmark Region	4,512	50–69	24	yes	no	14.9	–
Pornet (44)	France, 2004–2006	Database of the Association Mathilde, in charge of organizing BCS in Calvados; health insurance organizations database	4,865	50–74	24	yes	yes	44.3	Age of women, income level,
Larsen (45)	Denmark, 2008–2009	Central Denmark regional cancer screening administrative database; Danish Cancer Registry; Statistics Denmark; National Patient Register; National Pathology Data Bank	91,787	50–64	24	yes	no	20.2	–
Jensen (46)	Denmark, 2008–2009	Department for Public Health Programs database, Central Denmark Region; Statistics Denmark; Danish National Board of Health	13,288	50–69	24	yes	no	19.0	Gender of family physician
Wilf-Miron (47)	Israeli, 2006–2008	Maccabi Healthcare Services (MHS) computerized billing system; MHS computerized Performance Measurement System; Israeli Census for data on socio-economic status ranks and ethnicity	157,928	50–74	24	yes	yes	31.2	Age of women, income level
Roder (48)	Australia, 2001–2005	Australian BreastScreen program database; Australian Institute of Health and Welfare database	5,366,983	50–69	24	yes	no	44.9	–
Tavasoli (49)	Canada, 2013–2015	Integrated Client Management System database for cancer screening program; Ontario Health Insurance Plan’s Claims History databases; Ontario Cancer Registry and Pathology Information Management System; Client Agency Program Enrolment database and Corporate Providers Database; Canadian Institute of Health Information Discharge Abstract Database and National Ambulatory Care Reporting System	1,173,456	52–69	24	yes	no	47.6	Age of women, income level, place of residence, family number of comorbidities, gender of physician
Vermeer (50)	The Netherlands, 2007–2008	Database of regional screening organizations	1,279,982	50–75	24	yes	yes	18.0	Immigration status
O’Reilly (51)	UK, 2001–2004	Northern Ireland Quality Assurance Reference Centre; database of the Northern Ireland Longitudinal Study	37,059	48–64	36	yes	no	24.9	Age of women, place of residence, education level, number of comorbidities, marital status
Shin (52)	Korea, 2014–2015	Korean National Health Information Database	6,283,623	≥40	24	no	no	40.9	Income level, place of residence
Viuff (53)	Denmark, 2007–2010	The Danish Quality Database for Mammography Screening; The Danish National Patient Registry	650,003	50–69	24	yes	no	20.2	Age of women, number of comorbidities

^*^A full list of all the determinants reported by each study is shown in **Table S2**.

The risk of bias of the included studies is presented in **Table S2**. Requirements that were not satisfied were found for: sample size justification was unclear or missing in 10.3% of the studies; no measures were undertaken to address non-responders in 6.8% of the studies; basic data were not adequately described in 20.7% of the studies; limitations of the study were not discussed in 20.7% of the studies; sources of funding and conflicts of interest were not indicated in 6.8% of the studies, and ethical approval or consent of participants was not indicated in 17.2% of the studies.

### Pooled Estimates of the Determinants of Screening Non-Participation

Of all the 24 identified determinants (**Tables 1** and **S3**), nine were included for the meta-analysis. The other determinants were reported by less than three studies or had inconsistent definition were not meta-analyzed. The characteristics of the studies that reported these determinants are described in **Table 1**. All the determinates reported by the included studies are reported in **Table S3**. Having low income (OR: 1.20, 95% CI: 1.10–1.30), being in younger age (OR: 1.09, 95% CI: 1.01–1.18), having low education (OR: 1.18, 95% CI: 1.05–1.32), living far from an assigned screening unit (OR: 1.15, 95%CI: 1.07-1.24), being unmarried (OR: 1.68, 95% CI: 1.32–2.14), being an immigrant (OR: 2.64, 95% CI: 2.48–2.82), and having a male family physician (OR: 1.43, 95% CI: 1.20–1.61) was associated with a higher non-participation in screening (**Table 2** and **Figures S1–S5**).

**Table 2:** Summary of determinants of screening non-participation in breast cancer screening programs.

Determinants^a^	Number of studies	Number of women^b^	Non-participation %	Odds ratio	95% CI	I^2%^
**Income level**	14	12,500,262	32.7			99.6
high^c^		1,42,962	11.9–49.7	1.00	–	
Low		4,804,875	12.0–51.1	1.20	1.10–1.30	
**Age of women**	14	5,721,776	31.5			99.8
old		1,060,746	8.0–53.3	1.00	–	
young^d^		4,545,696	12.6–52.0	1.09	1.01–1.18	
**Place of residence**	7	9,342,846	27.9			99.5
rural^e^		528,624	12.4–51.3	1.00	–	
urban		2,545,607	11.9–45.9	1.01	0.90–1.12	
**Number of comorbidities**	6	2,412,969	22.6			99.5
Zero		2,101,610	12.0–51.7	1.00	–	
at least one		423,951	11.0–46.4	1.04	0.84–1.28	
**Education level**	5	1,160,622	24.6			90.6
high		73,651	19.8–29.0	1.00	–	
low ^f^		951,464	21.1–25.1	1.18	1.05–1.32	
**Distance to an assigned screening unit**	5	1,186,680	43.6			94.5
small^g^		549,621	18.0–54.0	1.00	–	
large		538,237	20.1–47.6	1.15	1.07–1.24	
**Marital status**	5	1,160,622	23.5			99.4
married^h^		620,694	17.3–22.0	1.00	–	
unmarried		134,188	31.1–35.0	1.68	1.32–2.14	
**Immigration status**	3	2,310,177	20.5			95.9
non-immigrants		2,210,697	15.7–25.0	1.00	–	
immigrants^i^		99,480	34.3–49.0	2.64	2.48–2.82	
**Gender of family physician**	3	2,272,225	24.9			98.6
Female		949,434	12.7–29.0	1.00	–	
Male		1,322,791	11.4–37.0	1.43	1.20–1.61	

a The first group of each determinant was the reference group.

b For each determinant, the total number of women is larger than the sum of women in the stratified groups, because there are studies that only provided the effect size of a determinant without the cross-tables behind it.

c The definition of high-income level varied in the included studies: “Most affluent 20%”, “most affluent 30%” and “most affluent 50% and above” was applied in 8, 2, and 4 studies, respectively. The heterogeneity related to the different definition of high income was explored in the stratified analyses.

d The definition of old age varied in the included studies: “60–64”, “60–69”, “67–69”, “65–70” and “70–74” was applied in 1, 1, 1, 6. and 5 studies, respectively. The heterogeneity related to the different definition of old age was explored in the stratified analyses.

e The definition of urban area was based on the population size in which the rural area was defined as area with less than 2,250 population in studies from UK. While the specific population size was not reported in studies from Canada and South Korea, the heterogeneity related to the different definition of rural area was explored in the stratified analyses.

f The definition of low education level varied in the included studies: “<Secondary graduate”, “≤10 years education” and “<University graduate” were applied in 1, 2, and 2 studies, respectively. The heterogeneity related to the different definition of low education was explored in the stratified analyses.

g The definition of small distance varied in included studies: “≤2.5 km”,” ≤5 km”,: “≤10 km”, and “≤20 km”, were applied in 1, 1, 1, and 2 studies, respectively. The heterogeneity related to the different definition of small distance was explored in the stratified analyses.

h Married woman was defined as woman married or living with a partner.

i Immigrant were defined as woman born abroad and both her two parents and four grandparents were born abroad.

### Stratified Analysis and Source of Heterogeneity

Substantial heterogeneity was found among the studies that reported the above-noted nine determinants. The Index of Inconsistency (I^2^) ranged from 90.6 to 99.8% for the studies which reported the education level and reported the age of women, respectively (**Table 2**).

In the stratified analysis the heterogeneity decreased for the resident place when stratified by whether or not a reminder was sent to non-attendees. When there was no reminder for non-attendees, women living in an urban area showed a higher non-participation than those living in a rural area (OR: 1.14, 95% CI: 1.03–1.26). However, when a reminder was sent, women living in an urban area showed a lower non-participation than those living in a rural area (OR: 0.83, 95% CI: 0.82–0.84) (**Table S4** and **Figure S6**). For education level, distance to an assigned screening unit, and marital status, whether a reminder was sent to non-attendees or not partly explained the heterogeneity across the studies, where the heterogeneity decreased in the stratified analysis (**Table S4**).

For income level, number of comorbidities, and marital status reporting adjusted estimate or not in the included studies partly explained the heterogeneity across the studies, where in these stratified groups the heterogeneity decreased (**Table S4**). The heterogeneity of the dichotomized determinants: age of women, education level, and distance to an assigned screening unit were partly explained by the different categorization of determinants. For example, the heterogeneity of the education level decreased from 90.6% for the overall estimate to 78.6% in the stratified group that defined ≤10 years education as low education (**Tables 2** and **S4**). However, the heterogeneity in almost all stratified groups with different categorization of determinants remained above a substantial level (I^2^ >50%).

### Sensitivity Analysis and Publication Bias

The pooled estimates of the determinants of screening participation were robust in the sensitivity analysis. The direction of the pooled estimates did not change when a single study was excluded sequentially (**Figure S7**). Publication bias was assessed for income and age of women. The Begg’s test of the asymmetry of the funnel plot did not reach statistical significance P = 0.743 and 0.661, respectively (**Figures S8, S9**).

## Discussion

### Main Results of This Review

In this meta-analysis excluding studies with self-reported data on non-participation in screening and/or studies on the non-participation in opportunistic screening, we found that lower income, younger age, lower education, living at a larger distance from an assigned screening unit, being unmarried, being an immigrant, and having a male family physician were associated with a higher non-participation in BCSPs. Women living in urban areas have higher non-participation in screening than women living in rural areas; however women living in urban areas have lower non-participation in screening when a reminder was sent to non-attenders. The heterogeneity of the pooled estimates was partially explained by whether or not a reminder was sent to non-attenders and whether or not the adjusted estimates were used.

### Comparison With Published Studies

Compared with other meta-analyses that included non-participation data from opportunistic screening and/or self-reported data, we found significant yet less strong association estimates with a narrower 95% CI for the well-known determinants of non-participation in screening. In our study, low-income women were more likely to not participate in a BCSP than high-income women (OR: 1.20, 95% CI: 1.10–1.30), whereas a meta-analysis reported a larger effect size with a wider 95% CI of low-income on non-participation in screening (OR: 1.35, 95% CI: 1.22–1.49) (12). Low educated women were more likely to not participate in a BCSP than high educated women. The effect size of low education on non-participation in screening was larger in a meta-analysis (OR: 1.61, 95% CI: 1.36–1.91) than our study (OR: 1.18, 95% CI: 1.05–1.32) (11), and the 95% CI was wider than our study. Immigrants were more likely to not participate in a BCSP than non-immigrants. The effect size of immigrant status on non-participation in screening was smaller in a meta-analysis (OR: 1.85, 95% CI: 1.27–2.70) than our study (OR: 2.64, 95% CI: 2.48–2.82) (12), but the 95% CI was wider than our study.

The main possible reasons for the difference between our estimates and the published meta-analyses are two-fold. First, the registry and self-reported data were mixed and pooled together in these reviews published thus far. As women tend to over-report the utilization of BC screening, the estimates in these reviews can be influenced by recall bias (18). Second, determinants of screening participation of a BCSP were not studied separately from an opportunistic screening in these reviews. However, a BCSP and an opportunistic screening have different implementation strategies (4), and can cover different women groups in a population (54), and have different determinants of non-participation in screening (55). We, however, focused on population-based BC screening programs with registry data, which can avoid the recall bias. The smaller 95% CIs indicate that we provided more accurate estimates.

Interestingly, when a reminder was not applied, women living in an urban area were more likely to not participate in screening than women living in a rural area (OR: 1.14, 95% CI: 1.03–1.26). However, when a reminder was sent, women living in an urban area were related to lower non-participate in screening (OR: 0.83, 95% CI: 0.82–0.84) than women living in rural area. A meta-analysis has shown that a reminder is effective in motivating more women to participate in a screening program (56). Our findings further suggest that the positive effect of a reminder plays a more important role in motivating women living in an urban area than women living in a rural area to attend a BCSP.

The pooled estimates for all the meta-analyzed determinants of non-participation in screening had substantial heterogeneity. Such heterogeneous estimates were also seen in other meta-analyses. For example, the I^2^ of two reviews on the effect of living in a rural area and comorbidity on BC screening participation was 95 and 99%, respectively (13, 57). In our stratified analysis a reminder sent to non-attendees or not and reporting adjusted estimate or not in the included studies partly explained the heterogeneity across the studies. Moreover, for the dichotomized determinants, since the contents/definitions of these determinants vary between the studies, pooled estimates are likely to be heterogeneous. In the stratified analyses, we found that the heterogeneity decreased slightly when studies were stratified based on the different categorization. As the results of the meta-analysis resembled that of the original studies, it suggest that despite of wide variation in the categorization of determinants, their impact to non-participation was similar in each study. However, we were not able to fully explain the heterogeneity. Other potential explanations could be the differences in study settings and methodologies of the included studies such as the different confounders that were adjusted for by different studies.

The study also has some limitations. First, only studies published in the English language were included; however, the publication bias was not statistically significant for the determinant income and age of women on screening non-participation. We would not expect a large difference between English or non-English publications for other determinants. Second, not all studies evaluated all nine determinants. Some determinants such as gender of family physician gender were only included in three studies. When a smaller number of studies are available, wider confidence intervals can be expected. Third, all the included studies were published from high-income countries where an organized breast cancer screening program was implemented. Moreover, half of the women included in the meta-analysis were of European or Canadian origin. Therefore, the results in this meta-analysis are less applicable to breast cancer screening globally. Lastly, the meta-analysis was based on data from the observational studies and most of the pooled ORs of the meta-analyzed determinants of non-participation in BC screening were below 2. Therefore, the determinants in our meta-analysis are less likely to be causally related to non-participation in BC screening.

### Conclusions

In this meta-analysis excluding studies focusing on opportunistic screening, or using self-reported data, women who were characterized by low income, younger age, low education, living at a large distance to an assigned screening unit, being unmarried, being an immigrant, and having a male family physician were associated with a high non-participation in a BCSP. Interventions to improve the participation of BCSP need to pay more attention to women that are characterized by the above-noted determinants. The association between these determinants and non-participation in BCSP screening was significant but less strong than the report from the reviews, namely, studies on the non-participation in opportunistic screening or with self-reported data on non-participation. This might be explained by a tendency of over-reporting screening utilization collected using a self-reporting method. This analysis only supports the relevance of studies with registry data of the non-participation in BCSP.

## Data Availability Statement

The original contributions presented in the study are included in the article/**Supplementary Material**. Further inquiries can be directed to the corresponding author.

## Author Contributions

LD: Conceptualization, systematic search strategy construction, paper selection and data extraction, data analysis, the original draft of manuscript writing and revision. JW: Second reader for paper selection and data extraction, manuscript reviewing, and editing. MJWG: Conceptualization, Methodology, Writing—Reviewing, Editing, and Validation. GHd: Supervision, Conceptualization, Methodology, Writing—Reviewing, Editing, and Validation. MG: Writing—Reviewing and Editing, Validation. GV: Conceptualization, Methodology, Writing—Reviewing, Editing, and Validation. All authors listed have made a substantial, direct, and intellectual contribution to the work and approved it for publication.

## Conflict of Interest

The authors declare that the research was conducted in the absence of any commercial or financial relationships that could be construed as a potential conflict of interest.

## Publisher’s Note

All claims expressed in this article are solely those of the authors and do not necessarily represent those of their affiliated organizations, or those of the publisher, the editors and the reviewers. Any product that may be evaluated in this article, or claim that may be made by its manufacturer, is not guaranteed or endorsed by the publisher.
