# Supplementary material for: Determinants of Non-Participation in Population-Based Breast Cancer Screening: A Systematic Review and Meta-Analysis
Source: Front Oncol. 2022 Mar 2;12:817222. doi: 10.3389/fonc.2022.817222 (PMC8924365; doi:10.3389/fonc.2022.817222)
Supplement: Supplementary file 1 [file DataSheet_1.docx]

## Supplementary Material

## PRISMA Checklist

| **Section/topic** | **#** | **Checklist item** | **Reported on page #** |
| --- | --- | --- | --- |
| **TITLE** | | |  |
| Title | 1 | Identify the report as a systematic review, meta-analysis, or both. | 1 |
| **ABSTRACT** | | |  |
| Structured summary | 2 | Provide a structured summary including, as applicable: background; objectives; data sources; study eligibility criteria, participants, and interventions; study appraisal and synthesis methods; results; limitations; conclusions and implications of key findings; systematic review registration number. | 2 |
| **INTRODUCTION** | | |  |
| Rationale | 3 | Describe the rationale for the review in the context of what is already known. | 3 |
| Objectives | 4 | Provide an explicit statement of questions being addressed with reference to participants, interventions, comparisons, outcomes, and study design (PICOS). | 3 |
| **METHODS** | | |  |
| Protocol and registration | 5 | Indicate if a review protocol exists, if and where it can be accessed (e.g., Web address), and, if available, provide registration information including registration number. | 3 |
| Eligibility criteria | 6 | Specify study characteristics (e.g., PICOS, length of follow-up) and report characteristics (e.g., years considered, language, publication status) used as criteria for eligibility, giving rationale. | 3-4 |
| Information sources | 7 | Describe all information sources (e.g., databases with dates of coverage, contact with study authors to identify additional studies) in the search and date last searched. | 3-4 |
| Search | 8 | Present full electronic search strategy for at least one database, including any limits used, such that it could be repeated. | Supplementary Material |
| Study selection | 9 | State the process for selecting studies (i.e., screening, eligibility, included in systematic review, and, if applicable, included in the meta-analysis). | 4 |
| Data collection process | 10 | Describe method of data extraction from reports (e.g., piloted forms, independently, in duplicate) and any processes for obtaining and confirming data from investigators. | 4 |
| Data items | 11 | List and define all variables for which data were sought (e.g., PICOS, funding sources) and any assumptions and simplifications made. | 4 |
| Risk of bias in individual studies | 12 | Describe methods used for assessing risk of bias of individual studies (including specification of whether this was done at the study or outcome level), and how this information is to be used in any data synthesis. | 4 |
| Summary measures | 13 | State the principal summary measures (e.g., risk ratio, difference in means). | 4 |
| Synthesis of results | 14 | Describe the methods of handling data and combining results of studies, if done, including measures of consistency (e.g., I^2^) for each meta-analysis. | 4-5 |

| **Section/topic** | **#** | **Checklist item** | **Reported on page #** |
| --- | --- | --- | --- |
| Risk of bias across studies | 15 | Specify any assessment of risk of bias that may affect the cumulative evidence (e.g., publication bias, selective reporting within studies). | 5 |
| Additional analyses | 16 | Describe methods of additional analyses (e.g., sensitivity or subgroup analyses, meta-regression), if done, indicating which were pre-specified. | 4-5 |
| **RESULTS** | | |  |
| Study selection | 17 | Give numbers of studies screened, assessed for eligibility, and included in the review, with reasons for exclusions at each stage, ideally with a flow diagram. | 5, 9 (Supplementary Material) |
| Study characteristics | 18 | For each study, present characteristics for which data were extracted (e.g., study size, PICOS, follow-up period) and provide the citations. | 5 |
| Risk of bias within studies | 19 | Present data on risk of bias of each study and, if available, any outcome level assessment (see item 12). | 5 and Supplementary Material |
| Results of individual studies | 20 | For all outcomes considered (benefits or harms), present, for each study: (a) simple summary data for each intervention group (b) effect estimates and confidence intervals, ideally with a forest plot. | 5, 10-14 and Supplementary Material |
| Synthesis of results | 21 | Present results of each meta-analysis done, including confidence intervals and measures of consistency. | 5, 15 |
| Risk of bias across studies | 22 | Present results of any assessment of risk of bias across studies (see Item 15). | 5 and Supplementary Material |
| Additional analysis | 23 | Give results of additional analyses, if done (e.g., sensitivity or subgroup analyses, meta-regression [see Item 16]). | 6 and Supplementary Material |
| **DISCUSSION** | | |  |
| Summary of evidence | 24 | Summarize the main findings including the strength of evidence for each main outcome; consider their relevance to key groups (e.g., healthcare providers, users, and policy makers). | 6-7 |
| Limitations | 25 | Discuss limitations at study and outcome level (e.g., risk of bias), and at review-level (e.g., incomplete retrieval of identified research, reporting bias). | 8 |
| Conclusions | 26 | Provide a general interpretation of the results in the context of other evidence, and implications for future research. | 8 |
| **FUNDING** | | |  |
| Funding | 27 | Describe sources of funding for the systematic review and other support (e.g., supply of data); role of funders for the systematic review. | 16 |

## Search strategy

**PubMed:**

("Breast Neoplasms" [Mesh] OR breast cancer* [tiab] OR breast tumo* [tiab] OR cancer of Breast [tiab] OR cancer of the Breast [tiab] OR breast carcinoma* [tiab] OR breast maligna* [tiab])

AND ("Mammography" [Mesh] OR mammogra* [tiab])

AND ("Mass screening" [Mesh] OR "Early Detection of Cancer" [Mesh] OR screening [tiab] OR early detection [tiab])

AND (attend* [tiab] OR complian* [tiab] OR uptak* [tiab] OR adher* [tiab] OR use [ti] OR usage [ti] OR participat* [tiab] OR non-participat* [tiab] OR rate [ti] OR rates [ti] OR screening behavio* [tiab] OR No-Show patients [Mesh] OR compliance [Mesh] OR facilities and services utilization [Mesh] OR social participation [Mesh] OR retention in care [Mesh] OR "Health Equity" [Mesh] OR "Healthcare Disparities" [Mesh] OR equity [tiab] OR inequity [tiab] OR equal* [tiab] OR inequal* [tiab])

AND ("Risk Factors" [Mesh] OR "Predictive Value of Tests" [Mesh] OR risk [tiab] OR predict* [tiab] OR deteminant* [tiab] OR factor* [tiab] OR relat* [ti] OR associat* [ti] OR "Population Characteristics" [Mesh] OR Socioeconomic factors [mesh])

**Web of Science:**

TS=((Breast cancer* OR Breast Tumor* OR Cancer of Breast OR Cancer of the Breast OR Breast Carcinoma* OR Breast Neoplasms OR breast maligna*) AND (Mammography OR mammogram*) AND (Screening OR Early detection OR Mass screening) AND (Attend* OR complian* OR adher* OR uptake OR facilities and services utilization OR Use OR usage OR participat* OR non-participat* OR social participation OR rate OR rates OR screening behavio* OR No-Show Patients OR retention in care OR Health Equity OR Healthcare Disparities OR equity OR inequity OR equal* OR inequal*) AND (Determinant* OR risk factor* OR predict* OR associat* OR relat* OR Population Characteristics OR Socioeconomic factor*))

**Embase:**

(‘Breast cancer*’/exp OR ‘Breast Tumor*’/exp OR ‘Cancer of Breast’/exp OR ‘Cancer of the Breast’/exp OR ‘Breast Carcinoma*’/exp OR ‘Breast Neoplasms’/exp OR ‘breast maligna*’:ab,ti)

AND (‘Mammography’/exp OR ‘mammogram’:ab,ti OR ‘mammograms’:ab,ti)

AND ('cancer screening'/exp OR 'mass screening'/exp OR 'mass radiography'/exp OR 'early cancer diagnosis'/exp)

AND ('patient attendance'/exp OR 'protocol compliance'/exp OR ‘Adhere*’:ab,ti OR 'health care utilization'/exp OR ‘Usage’:ab,ti OR 'refusal to participate'/exp OR ‘Retention’:ab,ti OR ‘No-Show Patients’:ab,ti OR ‘uptake’:ab,ti OR 'health equity'/exp OR 'health care disparity'/exp)

AND (‘Determinant*’/exp OR ‘risk factor*’/exp OR ‘predictor*’/exp OR 'epidemiology'/exp OR 'population and population related phenomena'/exp)

## Table S1: Items of the risk of bias assessment tool (AXIS)

| **Items** | **Content** |
| --- | --- |
| **Introduction** | |
| 1 | Were the aims/objectives of the study clear? |
| **Methods** | |
| 2 | Was the study design appropriate for the stated aim (s)? |
| 3 | Was the sample size justified? |
| 4 | Was the target/reference population clear defined? |
| 5 | Was the sample frame taken from an appropriate population base so that it closely represented the target/reference population under investigation? |
| 6 | Was the selection process likely to select subjects/participants that were representative of the target/reference population under investigation? |
| 7 | Were measures are undertaken to address and categories non-responders? |
| 8 | Were the risk factor and outcome variables measured appropriate to the aims of the study? |
| 9 | Were the risk factor and outcome variables measured correctly using instruments/measurements that had been trialed, piloted, or published previously? |
| 10 | Is it clear what was used to determining statistical significance and/or precision estimates? (e.g. p-values, confidence interval) |
| 11 | Were the methods (including statistical methods) sufficiently described to enable them to be repeated? |
| **Results** | |
| 12 | Were the basic data adequately described? |
| 13 | Does the response rate raise concerns about non-response bias? |
| 14 | If appropriate, was information about non-responders described? |
| 15 | Were the results internally consistent? |
| 16 | Were the results presented for all the analyses described in the methods? |
| **Discussion** | |
| 17 | Were the authors' discussion and conclusions justified by the results? |
| 18 | Where the limitations of the study discussed? |
| **Other** | |
| 19 | Were there any funding sources or conflicts of interest that may affect the authors' interpretation of the results? |
| 20 | Was the ethical approval or consent of participants attained? |

## Table S2 Quality assessment of the included studies

| Items | SS Hellmann, 2015 | M Vahabi, 2015 | RH Jack, 2014 | RR. Woods, 2018 | C Woodhead, 2016 | CL Price, 2010 | E Guillaume, 2017 | SN Vigod, 2011 | C Renshaw, 2010 | S Ouédraogo, 2014 | S St-Jacques, 2013 | LF Jensen, 2012 | M Le, 2019 | MN Zidar, 2015 | LF Jensen, 2015 | JT McDonald, 2017 | EM Berens, 2014 | LF Jensen, 2015 | LF Jensen, 2015 | C Pornet, 2010 | SH Larsen, 2018 | LF Jensen, 2012 | R Wilf-Miron, 2011 | D Roder, 2012 | SM Tavasoli, 2018 | B Vermeer, 2010 | Dermot O’Reilly, 2012 | DW Shin, 2020 | JH Viuff, 2020 |
| --- | --- | --- | --- | --- | --- | --- | --- | --- | --- | --- | --- | --- | --- | --- | --- | --- | --- | --- | --- | --- | --- | --- | --- | --- | --- | --- | --- | --- | --- |
| 1 | + | + | + | + | + | + | + | + | + | + | + | + | + | + | + | + | + | + | + | + | + | + | + | + | + | + | + | + | + |
| 2 | + | + | + | + | + | + | + | + | + | + | + | + | + | + | + | + | + | + | + | + | + | + | + | + | + | + | + | + | + |
| 3 | **?** | + | + | + | + | + | + | **?** | + | + | + | + | + | + | + | + | + | + | + | **-** | + | + | + | + | + | + | + | + | + |
| 4 | + | + | + | + | + | + | + | + | + | + | + | + | + | + | + | + | + | + | + | + | + | + | + | + | + | + | + | + | + |
| 5 | + | + | + | + | + | + | + | + | + | + | + | + | + | + | + | + | + | + | + | + | + | + | + | + | + | + | + | + | + |
| 6 | ? | + | + | + | + | + | + | ? | + | + | + | + | + | + | + | + | + | + | + | ? | + | + | + | + | + | + | + | + | + |
| 7 | - | + | + | N | + | N | N | - | N | N | N | N | N | N | N | N | N | N | N | N | N | N | N | N | N | N | N | N | N |
| 8 | + | + | + | + | + | + | + | + | + | + | + | + | + | + | + | + | + | + | + | + | + | + | + | + | + | + | + | + | + |
| 9 | + | + | - | + | + | + | + | + | + | + | + | + | + | + | + | + | + | + | + | + | + | + | + | + | + | + | + | + | + |
| 10 | + | + | + | + | + | + | + | + | + | + | + | + | + | + | + | + | + | + | + | + | + | + | + | + | + | + | + | + | + |
| 11 | + | + | + | + | + | + | + | + | + | + | + | + | + | + | + | + | + | + | + | + | + | + | + | + | + | + | + | + | + |
| 12 | + | + | + | + | + | + | + | + | + | + | + | + | + | + | + | - | - | + | + | + | + | + | - | - | + | - | + | - | + |
| 13 | ? | - | + | N | - | N | N | ? | N | N | N | N | N | N | N | N | N | N | N | N | N | N | N | N | N | N | N | N | N |
| 14 | - | + | + | N | - | N | N | - | N | N | N | N | N | N | N | N | N | N | N | N | N | N | N | N | N | N | N | N | N |
| 15 | + | + | + | + | + | + | + | + | + | + | + | + | + | + | + | + | + | + | + | + | + | + | + | - | + | + | + | + | + |
| 16 | + | - | + | + | + | + | + | + | + | + | + | + | + | + | + | + | + | + | + | + | + | + | + | - | + | + | + | + | + |
| 17 | + | + | + | + | + | + | + | ? | + | + | + | + | + | + | + | + | + | + | + | + | + | + | + | + | + | + | + | + | + |
| 18 | + | + | - | + | + | - | - | + | + | - | + | + | + | + | + | + | + | + | + | + | + | + | + | - | + | - | + | + | + |
| 19 | - | - | - | - | - | - | ? | - | - | - | - | - | - | - | - | - | - | - | - | - | - | - | - | ? | - | - | - | - | - |
| 20 | + | + | ? | N | N | ? | ? | + | N | + | + | N | + | + | N | + | + | N | N | ? | N | N | N | + | + | ? | + | + | N |

* Yes = +; No = -; Don’t know = ?; Not applicable = N

## Table S3: Summary of studies that reported the association between determinants and screening non-participation in a breast cancer screening program and studies included in the meta-analysis

| **Determinants** | **Studies reported the determinant** | **Studies included in the meta-analysis** |
| --- | --- | --- |
| Income level | M Vahabi ^26^, RH Jack ^27^ , RR. Woods ^28^ , E Guillaume ^31^ , C Renshaw ^33^ , S Ouédraogo ^34^ , S St-Jacques ^35^ , LF Jensen ^36^ , M Le ^37^ , JT McDonald ^40^ , LF Jensen ^43^ , C Pornet ^44^ , SH Larsen ^45^ , LF Jensen ^46^ , R Wilf-Miron ^47^ , SM Tavasoli ^49^ , Shin DW ^52^ | M Vahabi ^26^, RH Jack ^27^ , RR. Woods ^28^ , E Guillaume ^31^ , C Renshaw ^33^ , S Ouédraogo ^34^ , S St-Jacques ^35^ , LF Jensen ^36^ , M Le ^37^ , JT McDonald ^40^ , C Pornet ^44^ , R Wilf-Miron ^47^ , SM Tavasoli ^49^ , Shin DW ^52^ |
| Age of women | RR. Woods ^28^ , E Guillaume^31^ , C Renshaw ^33^ , S Ouédraogo ^34^ , S St-Jacques ^35^ , LF Jensen ^36^ , M Le ^37^ , MN Zidar ^38^ , LF Jensen ^39^ , EM Berens ^41^ , LF Jensen ^42^ , C Pornet ^44^ , SH Larsen ^45^ , LF Jensen ^46^ , R Wilf-Miron ^47^ , SM Tavasoli ^49^ , D O'Reilly ^51^ , JH Viuff ^53^ | RR. Woods ^28^ , E Guillaume^31^ , C Renshaw ^33^ , S Ouédraogo ^34^ , S St-Jacques ^35^ , M Le ^37^ , MN Zidar ^38^ , LF Jensen ^39^ , EM Berens ^41^ , C Pornet ^44^ , R Wilf-Miron ^47^ , SM Tavasoli ^49^ , D O'Reilly ^51^ , JH Viuff ^53^ |
| Place of residence | M Vahabi ^26^ , S Ouédraogo ^34^ , S St-Jacques ^35^ , JT McDonald ^40^ , SM Tavasoli ^49^ , D O'Reilly ^51^ , DW Shin ^52^ | M Vahabi ^26^ , S Ouédraogo ^34^ , S St-Jacques ^35^ , JT McDonald ^40^ , SM Tavasoli ^49^ , D O'Reilly ^51^ , DW Shin ^52^ |
| Number of comorbidities | RR. Woods ^28^ , SN Vigod ^32^ , LF Jensen ^39^ , LF Jensen ^42^ , SH Larsen ^45^ , SM Tavasoli ^49^ , D O'Reilly ^51^ , JH Viuff ^53^ | RR. Woods ^28^ , SN Vigod ^32^ , LF Jensen ^39^ , SM Tavasoli ^49^ , D O'Reilly ^51^ , JH Viuff ^53^ |
| Education level | SN Vigod ^32^ , LF Jensen ^36^ , M Le ^37^ , LF Jensen ^39^ , JT McDonald ^40^ , LF Jensen ^42^ , D O'Reilly ^51^ , SH Larsen ^45^ | SN Vigod ^32^ , M Le ^37^ , LF Jensen ^39^ , JT McDonald ^40^ , D O'Reilly ^51^ |
| Distance to an assigned screening unit | E Guillaume ^31^ , S St-Jacques ^35^ , LF Jensen ^36^ , MN Zidar ^38^ , JT McDonald ^40^ , LF Jensen ^46^ | E Guillaume ^31^ , S St-Jacques ^35^ , LF Jensen ^36^ , MN Zidar ^38^ , JT McDonald ^40^ |
| Marital status | SN Vigod ^32^ , LF Jensen ^36^ , M Le ^37^ , LF Jensen ^39^ , JT McDonald ^40^ , SH Larsen ^45^ , LF Jensen ^46^ , D O'Reilly ^51^ | SN Vigod ^32^ , M Le ^37^ , LF Jensen ^39^ , JT McDonald ^40^ , D O'Reilly ^51^ |
| Immigration status | LF Jensen ^36^ , M Le ^37^ , LF Jensen ^39^ , SH Larsen ^45^ , LF Jensen ^46^ , B Vermeer ^50^ | LF Jensen ^36^ , M Le ^37^ , B Vermeer ^50^ |
| Physician's gender | M Vahabi ^26^ , LF Jensen ^46^ , SM Tavasoli ^49^ | M Vahabi ^26^ , LF Jensen ^46^ , SM Tavasoli ^49^ |
| Body mass index | SS Hellmann ^25^ | - |
| Mental disease | C Woodhead ^29^ , SN Vigod ^32^ | - |
| Type of insurance | S Ouédraogo ^34^ , C Pornet ^44^ , R Wilf-Miron ^47^ | * |
| Car access | LF Jensen ^36^ , D O'Reilly ^51^ | - |
| Travel time to the assigned screening unit | S Ouédraogo ^34^ | - |
| Net assest | M Le ^37^ | - |
| Employment status | LF Jensen ^36^ , M Le ^37^ , D O'Reilly ^51^ | * |
| Disability status | M Le ^37^ , DW Shin ^52^ | - |
| Self-assessed health status | M Vahabi ^26^ , LF Jensen ^42^ , D O'Reilly ^51^ | * |
| Residential ownership | D O'Reilly ^51^ , LF Jensen ^36^ | - |
| Social support | LF Jensen ^43^ | - |
| Cancer screening attending experience | RR. Woods ^28^ , CL Price ^30^ , SH Larsen ^45^ | * |
| Cancer screening invitation experience | C Renshaw ^33^ | - |
| Ethnicity | RH Jack ^27^ , CL Price ^30^ , C Renshaw ^33^ , D Roder ^48^ | * |
| Number of primary care visits | RR. Woods ^28^ , SN Vigod | - |

-: Determinants that were reported by less than three studies.

*: Determinants that were measure or defined differently that not be able to be meta-analyzed.

## Table S4 Stratified analyses for the determinants of screening non-participation in breast cancer screening programs

| **Determinants** | **Variable** | **Stratified groups** | **N^a^** | **Non-participation %** | **OR** | **95%CI** | **I^2^%** |
| --- | --- | --- | --- | --- | --- | --- | --- |
| **Income**  **Level**  **(low vs.**  **high (ref))** | Type of invitation | Any invitation | 10 | 31.2-49.7 | 1.22 | 1.10-1.35 | 99.7 |
|  |  | The first invitation | 4 | 26.0-49.9 | 1.13 | 0.99-1.30 | 98.2 |
|  | Reference income group | Most affluent 20% | 8 | 36.0-49.9 | 1.19 | 1.08-1.31 | 99.7 |
|  |  | Most affluent 30% | 2 | 21.1-47.5 | 1.45 | 0.88-2.39 | 98.8 |
|  |  | Most affluent 50% and above | 4 | 26.0-45.0 | 1.09 | 1.00-1.18 | 94.0 |
|  | Study region | North America | 4 | 36.0-49.7 | 1.16 | 1.04-1.31 | 99.7 |
|  |  | Europe | 8 | 21.1-49.9 | 1.23 | 1.05-1.45 | 99.9 |
|  |  | Asia | 2 | 31.2-40.9 | 1.13 | 1.10-1.16 | 0.0 |
|  | Screening interval | 24 months | 12 | 21.1-49.9 | 1.17 | 1.08-1.27 | 99.6 |
|  |  | 36 months | 2 | 37.9-39.0 | 1.36 | 1.21-1.53 | 98.0 |
|  | Payment of screening | Free | 12 | 21.1-49.9 | 1.22 | 1.12-1.34 | 99.7 |
|  |  | Co-payment | 2 | 26.0-40.9 | 1.02 | 1.00-1.04 | 0.0 |
|  | Reminder for non-attenders | Yes | 8 | 26.0-49.9 | 1.13 | 1.03-1.24 | 99.3 |
|  |  | No | 6 | 21.1-47.6 | 1.29 | 1.11-1.49 | 99.8 |
|  | Adjusted estimate | Yes | 6 | 31.2-49.9 | 1.13 | 1.11-1.16 | 0.0 |
|  |  | No | 8 | 21.1-49.7 | 1.24 | 1.11-1.38 | 99.8 |
| **Woman’s age**  **(young vs. old (ref))** | Type of invitation | Any invitation | 12 | 19.0-50.8 | 1.09 | 0.99-1.19 | 99.8 |
|  |  | The first invitation | 2 | 26.0-49.9 | 1.15 | 1.06-1.25 | 63.5 |
|  | Reference age group | 60-64 | 1 | 24.9 | 1.30 | 1.04-1.62 | - |
|  |  | 60-70 | 1 | 49.7 | 1.27 | 1.25-1.28 | - |
|  |  | 65-70 | 6 | 20.2-50.8 | 0.99 | 0.91-1.08 | 99.6 |
|  |  | 67-69 | 1 | 47.6 | 1.63 | 1.61-1.65 | - |
|  |  | 70-74 | 5 | 19.0-49.9 | 1.06 | 0.98-1.15 | 90.0 |
|  | Study region | North America | 3 | 47.6-49.7 | 1.29 | 1.01-1.65 | 99.9 |
|  |  | Europe | 10 | 19.0-50.8 | 1.04 | 0.96-1.13 | 99.2 |
|  |  | Asia | 1 | 31.2 | 1.00 | 1.00-1.01 | - |
|  | Screening interval | 24 months | 12 | 19.0-50.8 | 1.10 | 1.00-1.20 | 99.9 |
|  |  | 36 months | 2 | 24.9-37.9 | 1.08 | 0.79-1.49 | 87.7 |
|  | Payment of screening | Free | 12 | 19.0-26.0 | 1.10 | 1.00-1.21 | 99.8 |
|  |  | Co-payment | 2 | 20.2-50.8 | 1.04 | 0.81-1.34 | 99.1 |
|  | Reminder for non-attenders | Yes | 7 | 19.0-50.8 | 1.14 | 1.04-1.24 | 99.8 |
|  |  | No | 7 | 21.5-49.9 | 1.05 | 0.86-1.29 | 99.9 |
|  | Adjusted estimate | Yes | 5 | 24.9-49.9 | 1.07 | 0.94-1.21 | 99.9 |
|  |  | No | 9 | 19.0-50.8 | 1.07 | 1.01-1.29 | 86.6 |
| **Place of residence**  **(urban vs. rural (ref))** | Type of invitation | Any invitation | 6 | 24.9-47.9 | 1.04 | 0.92-1.17 | 99.6 |
|  |  | The first invitation | 1 | 45.0 | 0.83 | 0.79-0.87 | - |
|  | Study region | North America | 4 | 36.0-47.9 | 0.91 | 0.80-1.03 | 99.6 |
|  |  | Europe | 2 | 24.9-47.5 | 1.16 | 0.66-2.03 | 99.1 |
|  |  | Asia | 1 | 40.9 | 1.14 | 1.10-1.17 | - |
|  | Screening interval | 24 months | 6 | 36.0-47.9 | 0.94 | 0.84-1.04 | 99.5 |
|  |  | 36 months | 1 | 24.9 | 1.54 | 1.45-1.64 | - |
|  | Payment of screening | Free | 6 | 24.9-47.9 | 0.99 | 0.88-1.11 | 99.5 |
|  |  | Co-payment | 1 | 40.9 | 1.14 | 1.10-1.17 | - |
|  | Reminder for non-attenders | Yes | 3 | 45.0-47.9 | 0.83 | 0.82-0.84 | 0.0 |
|  |  | No | 4 | 24.9-47.6 | 1.14 | 1.03-1.26 | 99.0 |
|  | Adjusted estimate | Yes | 4 | 24.9-47.5 | 1.06 | 0.83-1.35 | 98.9 |
|  |  | No | 3 | 36.0-47.9 | 0.94 | 0.81-1.09 | 99.8 |
| **Number of**  **comorbidities**  **(at least one vs. zero (ref))** | Comorbidity measurement | CCI ^b^ | 2 | 20.2-47.6 | 0.99 | 0.79-1.25 | 84.3 |
|  |  | ADG ^c^ | 1 | 49.7 | 0.81 | 0.80-0.82 | - |
|  |  | Number of conditions | 3 | 24.9-39.2 | 1.16 | 0.79-1.71 | 98.6 |
|  | Type of  invitation | Any invitation | 6 | 20.2-49.7 | 1.04 | 0.84-1.28 | 99.5 |
|  |  | The first invitation | 0 | - | - | - | - |
|  | Study region | North America | 3 | 39.2-49.7 | 0.86 | 0.78-0.95 | 97.0 |
|  |  | Europe | 3 | 20.2-24.9 | 1.21 | 0.85-1.73 | 98.6 |
|  | Screening interval | 24 months | 5 | 20.2-49.7 | 1.05 | 0.83-1.33 | 99.6 |
|  |  | 36 months | 1 | 24.9 | 0.99 | 0.93-1.06 | - |
|  | Payment of  screening | Free | 6 | 20.2-49.7 | 1.04 | 0.84-1.28 | 99.5 |
|  |  | Co-payment | 0 | - | - | - | - |
|  | Reminder for  non-attenders | Yes | 1 | 49.7 | 0.81 | 0.80-0.82 | - |
|  |  | No | 5 | 20.2-47.6 | 1.10 | 0.81-1.48 | 99.3 |
|  | Adjusted estimate | Yes | 3 | 20.2-39.2 | 1.01 | 0.94-1.08 | 5.3 |
|  |  | No | 3 | 21.1-49.7 | 1.05 | 0.78-1.40 | 99.8 |
| **Education level**  **(low vs.**  **high (ref))** | Type of invitation | Any invitation | 3 | 21.1-39.2 | 1.08 | 0.98-1.17 | 71.3 |
|  |  | The first invitation | 2 | 26.0-45.0 | 1.31 | 1.24-1.38 | 0.0 |
|  | Reference education level | < Secondary graduate | 1 | 39.2 | 1.35 | 1.04-1.76 | - |
|  |  | ≤ 10 years education | 2 | 21.1-26.0 | 1.17 | 0.99-1.37 | 78.6 |
|  |  | < University graduate | 2 | 24.9-45.0 | 1.15 | 0.88-1.49 | 96.7 |
|  | Study region | North America | 2 | 39.2-45.0 | 1.31 | 1.24-1.39 | 0.0 |
|  |  | Europe | 3 | 21.1-26.0 | 1.10 | 1.00-1.20 | 79.1 |
|  | Screening interval | 24 months | 4 | 21.1-45.0 | 1.24 | 1.08-1.42 | 90.8 |
|  |  | 36 months | 1 | 24.9 | 1.00 | 0.93-1.08 | - |
|  | Payment of screening | Free | 4 | 21.1-45.0 | 1.15 | 1.02-1.31 | 92.3 |
|  |  | Co-payment | 1 | 26.0 | 1.29 | 1.11-1.50 | - |
|  | Reminder for non-attenders | Yes | 2 | 26.0-45.0 | 1.31 | 1.24-1.38 | 0.0 |
|  |  | No | 3 | 21.1-39.2 | 1.08 | 0.98-1.17 | 71.3 |
|  | Adjusted estimate | Yes | 3 | 24.9-45.0 | 1.19 | 0.96-1.48 | 93.6 |
|  |  | No | 2 | 21.1-26.0 | 1.17 | 0.99-1.37 | 78.6 |
| **Distance to an assigned screening unit**  **(large vs. small (ref))** | Type of invitation | Any invitation | 2 | 19.0-47.9 | 1.12 | 1.04-1.21 | 95.5 |
|  |  | The first invitation | 3 | 45.0-49.9 | 1.24 | 0.84-1.83 | 95.7 |
|  | Reference distance level | ≤ 2.5 km | 1 | 47.9 | 1.06 | 1.05-1.07 | - |
|  |  | ≤ 5 km | 1 | 49.9 | 1.51 | 1.33-1.72 | - |
|  |  | ≤ 10 km | 1 | 45.0 | 1.02 | 0.93-1.12 | - |
|  |  | ≤ 20 km | 2 | 19.0-21.1 | 1.16 | 1.13-1.19 | 0.0 |
|  | Study region | North America | 2 | 45.0-47.9 | 1.06 | 1.05-1.07 | 0.0 |
|  |  | Europe | 3 | 19.0-49.9 | 1.25 | 1.09-1.42 | 87.5 |
|  | Screening interval | 24 months | 5 | 19.0-49.9 | 1.15 | 1.07-1.24 | 94.5 |
|  |  | 36 months | 0 | - | - | - | - |
|  | Payment of screening | Free | 4 | 21.1-49.9 | 1.15 | 1.06-1.25 | 95.8 |
|  |  | Co-payment | 1 | 19.0 | 1.14 | 1.03-1.25 | - |
|  | Reminder for non-attenders | Yes | 3 | 45.0-49.9 | 1.17 | 0.98-1.39 | 93.3 |
|  |  | No | 2 | 19.0-21.1 | 1.16 | 1.13-1.19 | 0.0 |
|  | Adjusted estimate | Yes | 2 | 45.0-49.9 | 1.24 | 0.84-1.83 | 95.7 |
|  |  | No | 3 | 19.0-47.9 | 1.12 | 1.04-1.21 | 95.5 |
| **Marital status**  **(unmarried vs.**  **Married (ref))** | Type of invitation | Any invitation | 3 | 24.9-39.2 | 1.73 | 1.09-2.76 | 97.5 |
|  |  | The first invitation | 2 | 19.0-45.0 | 1.58 | 1.56-1.59 | 0.0 |
|  | Study region | North America | 2 | 39.2-45.0 | 1.72 | 1.37-2.16 | 0.0 |
|  |  | Europe | 3 | 19.0-26.0 | 1.66 | 1.24-2.22 | 99.7 |
|  | Screening interval | 24 months | 4 | 19.0-45.0 | 1.82 | 1.39-2.40 | 99.5 |
|  |  | 36 months | 1 | 24.9 | 1.26 | 1.11-1.43 | - |
|  | Payment of screening | Free | 4 | 19.0-45.0 | 1.72 | 1.17-2.51 | 96.4 |
|  |  | Co-payment | 1 | 26.0 | 1.58 | 1.56-1.59 | - |
|  | Reminder for non-attenders | Yes | 2 | 26.0-45.0 | 1.58 | 1.56-1.59 | 0.0 |
|  |  | No | 3 | 19.0-39.2 | 1.73 | 1.09-2.76 | 97.5 |
|  | Adjusted estimate | Yes | 3 | 24.9-45.0 | 1.49 | 1.17-1.90 | 63.5 |
|  |  | No | 2 | 19.0-26.0 | 1.89 | 1.32-2.70 | 99.8 |
| **Immigration status**  **(immigrant vs. non-immigrant (ref))** | Type of invitation | Any invitation | 1 | 18.0-21.1 | 2.54 | 2.10-3.07 | 97.8 |
|  |  | The first invitation | 2 | 26.0 | 2.81 | 2.76-2.85 | 0.0 |
|  | Study region | North America | 0 | - | - | - | - |
|  |  | Europe | 3 | 18.0-26.0 | 2.64 | 2.48-2.82 | 95.9 |
|  |  | Asia | 0 | - | - | - | - |
|  | Screening interval | 24 months | 3 | 18.0-26.0 | 2.64 | 2.48-2.82 | 95.9 |
|  |  | 36 months | 0 | - | - | - | - |
|  | Payment of screening | Free | 2 | 18.0-21.1 | 2.54 | 2.10-3.07 | 97.8 |
|  |  | Co-payment | 1 | 26.0 | 2.81 | 2.76-2.85 | - |
|  | Reminder for non-attenders | Yes | 2 | 18.0-26.0 | 2.80 | 2.77-2.83 | 0.0 |
|  |  | No | 1 | 21.1 | 2.30 | 2.18-2.43 | - |
|  | Adjusted estimate | Yes | 0 | - | - | - | - |
|  |  | No | 3 | 18.0-26.0 | 2.64 | 2.48-2.82 | 95.9 |
| **Physician’s gender**  **(female vs.**  **male (ref))** | Type of invitation | Any invitation | 2 | 36.0-47.6 | 1.56 | 1.33-1.85 | 99.1 |
|  |  | The first invitation | 1 | 19.0 | 1.08 | 0.97-1.20 | - |
|  | Study region | North America | 2 | 36.0-47.6 | 1.6 | 1.33-1.85 | 99.1 |
|  |  | Europe | 1 | 19.0 | 1.08 | 0.97-1.20 | - |
|  | Screening interval | 24 months | 3 | 19.0-47.6 | 1.41 | 1.20-1.61 | 98.6 |
|  |  | 36 months | 0 | - | - | - | - |
|  | Payment of screening | Free | 3 | 19.0-47.6 | 1.41 | 1.20-1.61 | 98.6 |
|  |  | Co-payment | 0 | - | - | - | - |
|  | Reminder for non-attenders | Yes | 0 | - | - | - | - |
|  |  | No | 3 | 19.0-47.6 | 1.41 | 1.20-1.61 | 98.6 |
|  | Adjusted estimate | Yes | 1 | 47.6 | 1.69 | 1.64-1.75 | - |
|  |  | No | 2 | 19.0-36.0 | 1.25 | 0.94-1.67 | 96.4 |

a: Number of studies; b: Charlson Comorbidity Index; c: Aggregated Diagnosis Groups


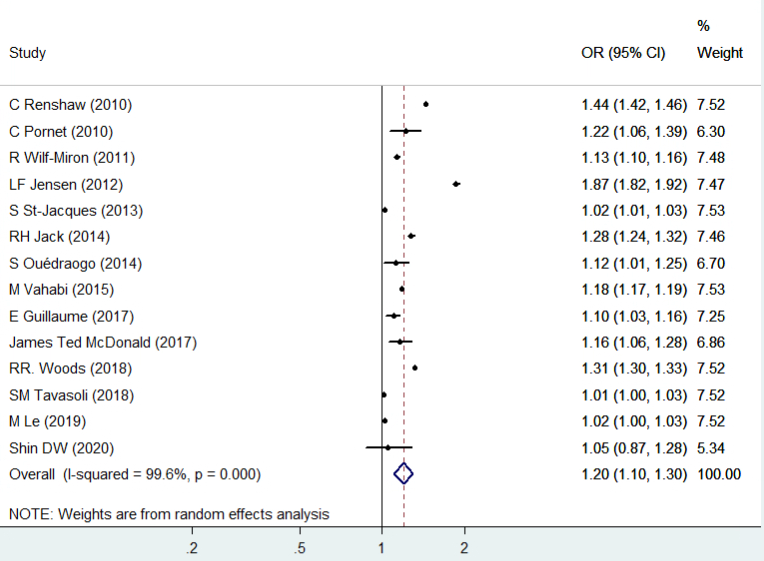


## Figure S1 Forest plot of the association between income level (low vs. high) and screening non-participation of breast cancer screening programs.


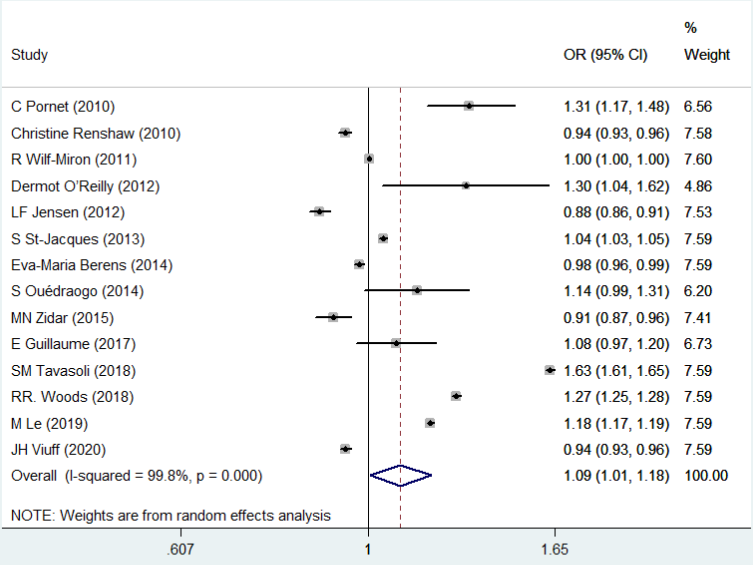


## Figure S2 Forest plot of the association between women’s age (young vs. old) and screening non-participation of breast cancer screening programs.


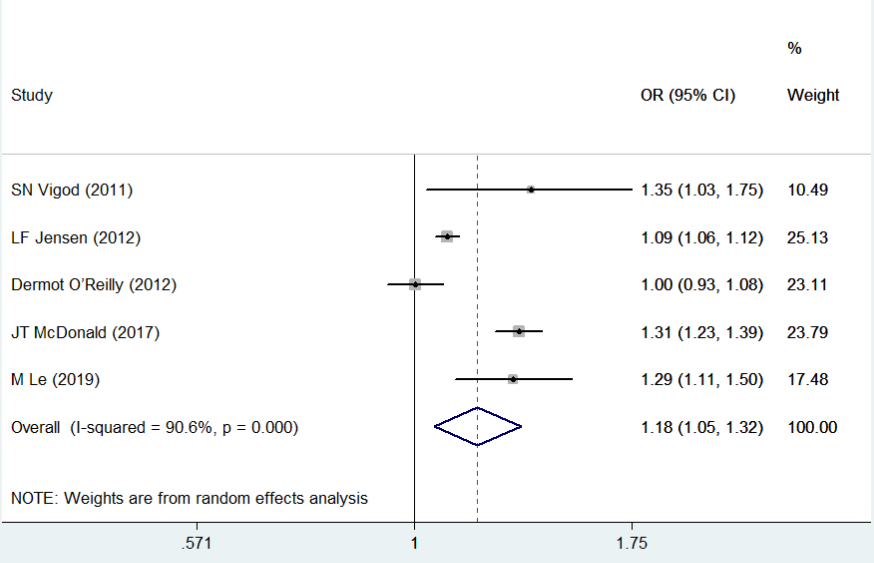


## Figure S3 Forest plot of the association between education level (low vs. high) and screening non-participation of breast cancer screening programs.


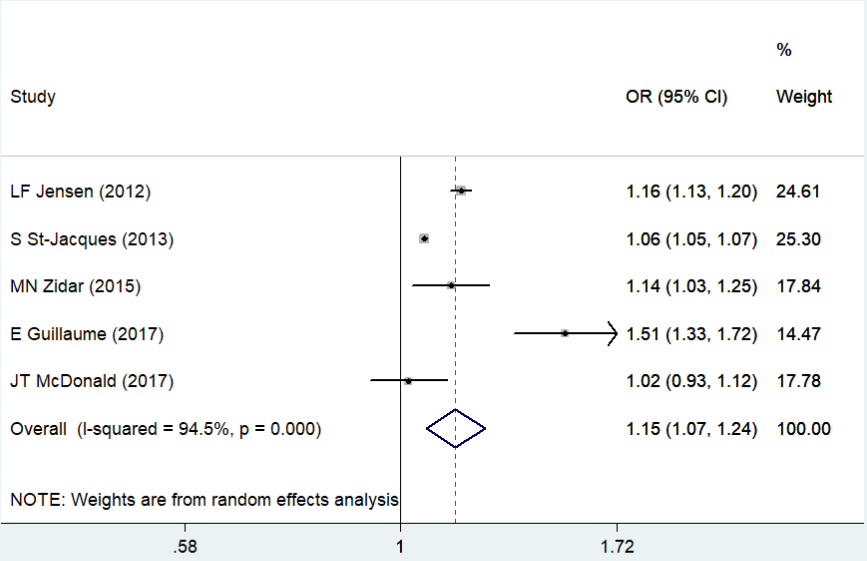


Figure S4 Forest plot of the association between women’s living distance to an assigned screening unit (large vs. small) and screening non-participation of breast cancer screening programs.


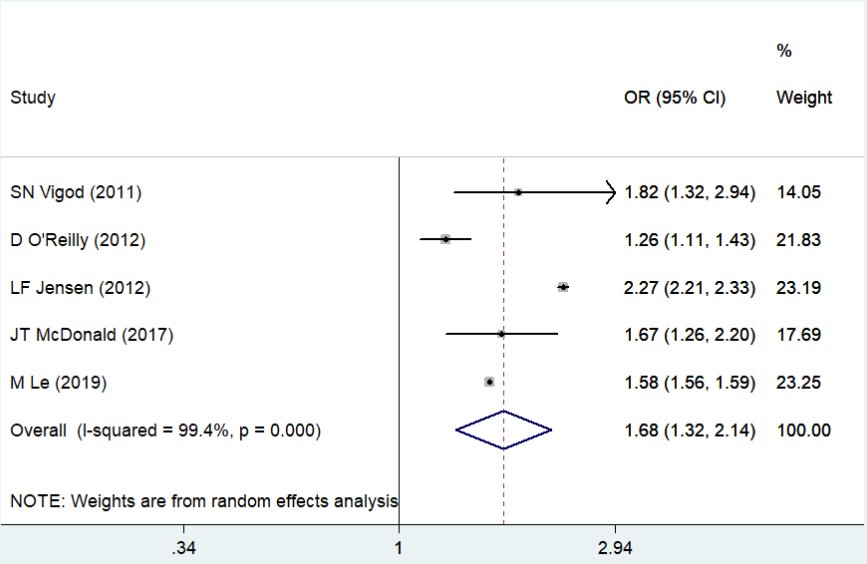


## Figure S5 Forest plot of the association between women’s marital status (unmarried vs. married) and screening non-participation of breast cancer screening programs.


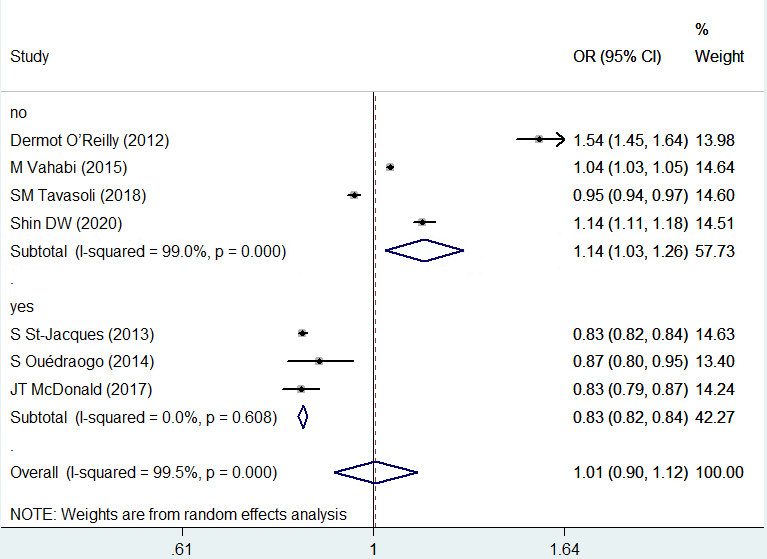


## Figure S6 Forest plot of the association between place of residence (urban vs. rural) and screening non-participation of breast cancer screening programs stratified by reminders sent to non-attendees yes/no.


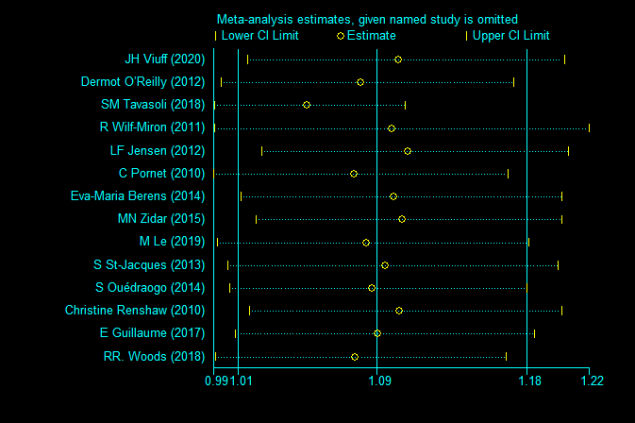

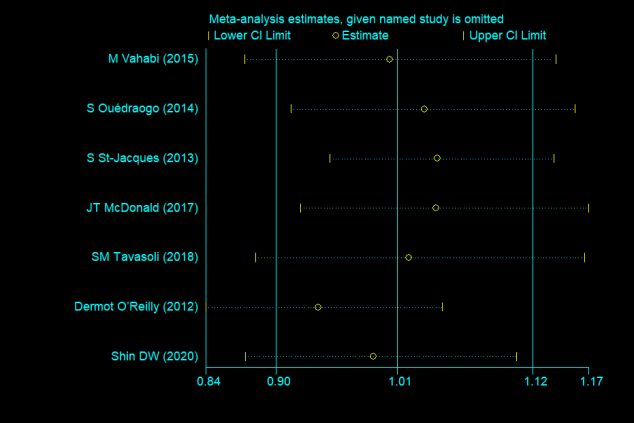

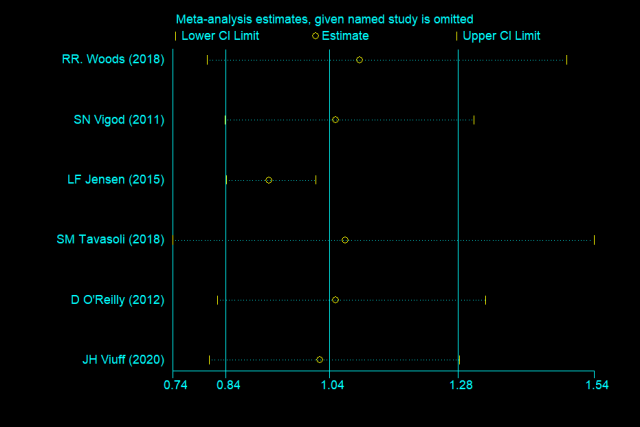

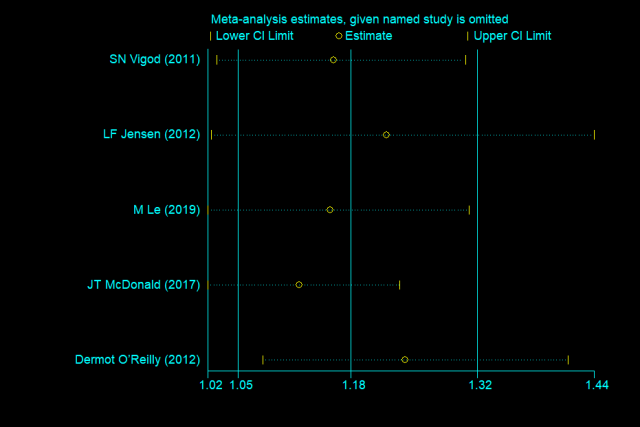

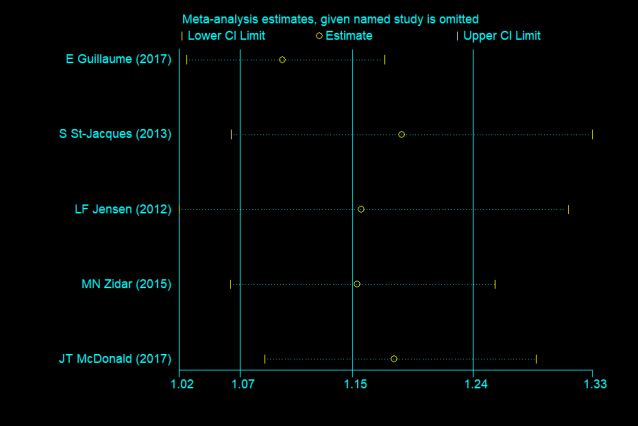

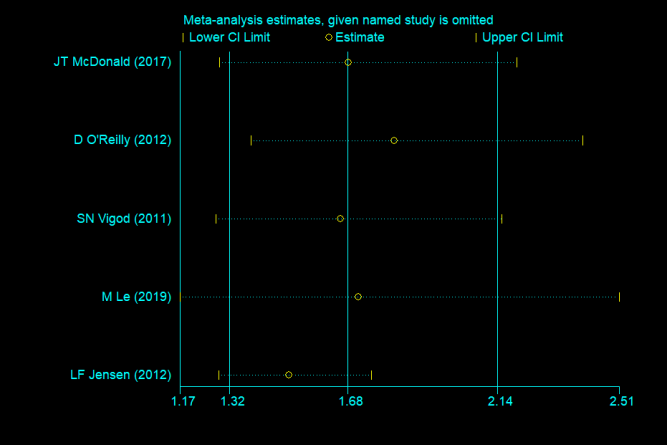

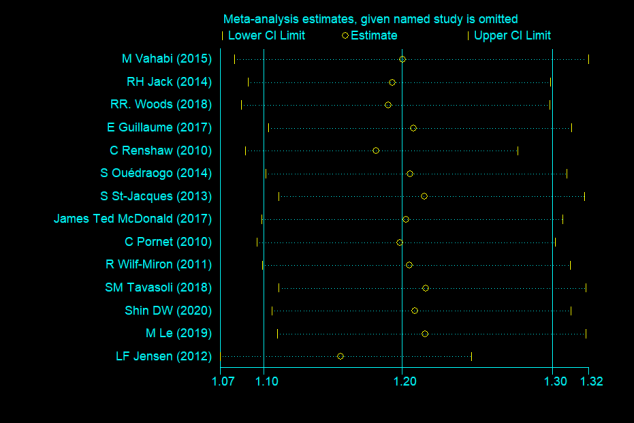


**A**

**B**

**C**

**D**

**E**

**F**

**G**

**H**

**I**

## Figure S7 Sensitivity analysis for the determinants of screening non-participation in breast cancer screening programs (A: income level, B: Age of women, C: Place of residence, D: Number of comorbidities, E: education level, F: Distance to assigned screening unit, G: marital status)


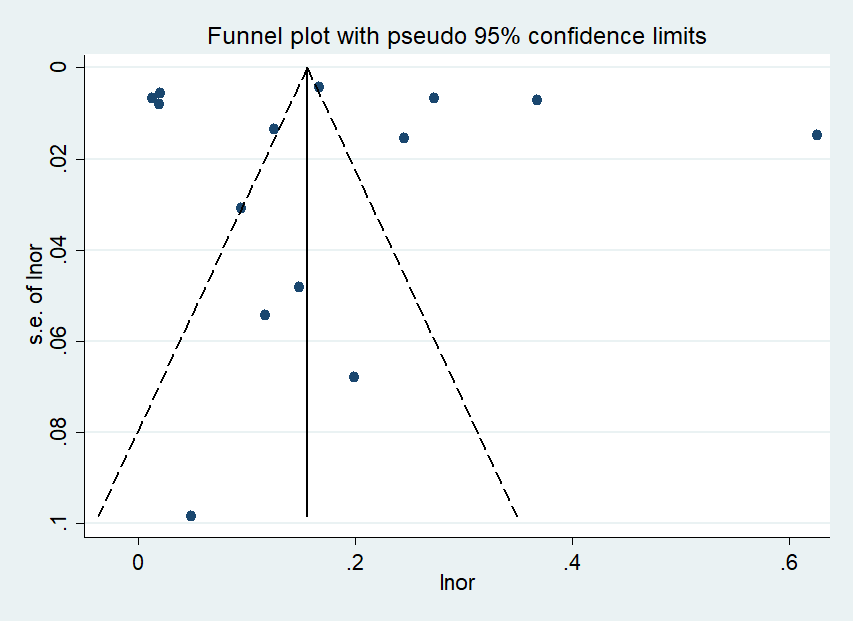


## Figure S8 Funnel plot of the included studies on the association between women’s income level and screening non-participation of breast cancer screening programs.


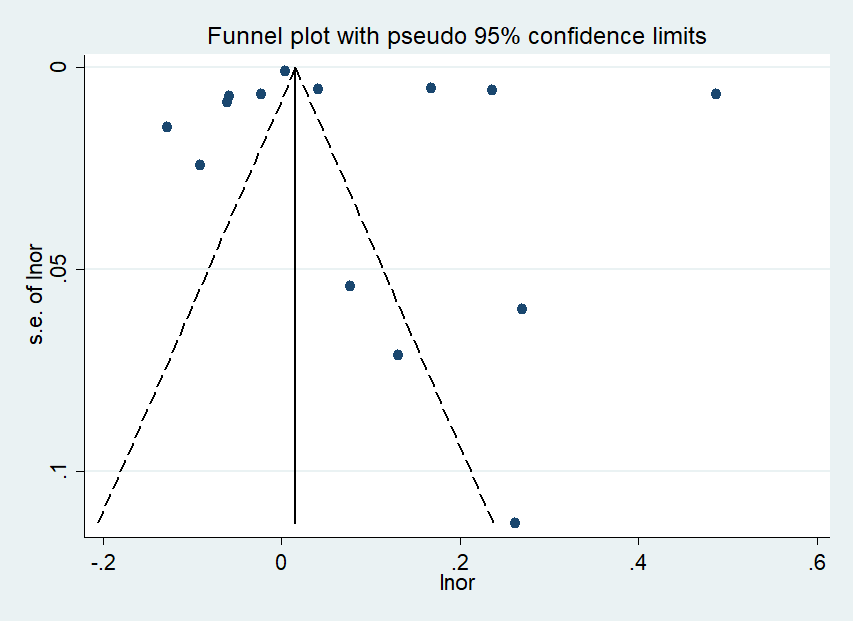


## Figure S9 Funnel plot of the included studies on the association between women’s age and screening non-participation of breast cancer screening programs.
